# The importance of educational tools and a new software solution for visualizing and quantifying report correction in radiology training

**DOI:** 10.1038/s41598-024-51462-4

**Published:** 2024-01-12

**Authors:** Luca Salhöfer, Johannes Haubold, Maurice Gutt, René Hosch, Lale Umutlu, Mathias Meetschen, Maximilian Schuessler, Michael Forsting, Felix Nensa, Benedikt Michael Schaarschmidt

**Affiliations:** 1 Institute of Diagnostic and Interventional Radiology and Neuroradiology, University Hospital Essen, Hufelandstr. 55, 45147 Essen, Germany; 2 Institute for Artificial Intelligence in Medicine, University Hospital Essen, Essen, Germany; 3 Central IT Services, University Hospital Essen, Essen, Germany

**Keywords:** Translational research, Software, Diagnosis, Medical imaging

## Abstract

A novel software, DiffTool, was developed in-house to keep track of changes made by board-certified radiologists to preliminary reports created by residents and evaluate its impact on radiological hands-on training. Before (t_0_) and after (t_2−4_) the deployment of the software, 18 residents (median age: 29 years; 33% female) completed a standardized questionnaire on professional training. At t_2−4_ the participants were also requested to respond to three additional questions to evaluate the software. Responses were recorded via a six-point Likert scale ranging from 1 (“strongly agree”) to 6 (“strongly disagree”). Prior to the release of the software, 39% (7/18) of the residents strongly agreed with the statement that they manually tracked changes made by board-certified radiologists to each of their radiological reports while 61% were less inclined to agree with that statement. At t_2−4_, 61% (11/18) stated that they used DiffTool to track differences. Furthermore, we observed an increase from 33% (6/18) to 44% (8/18) of residents who agreed to the statement “I profit from every corrected report”. The DiffTool was well accepted among residents with a regular user base of 72% (13/18), while 78% (14/18) considered it a relevant improvement to their training. The results of this study demonstrate the importance of providing a time-efficient way to analyze changes made to preliminary reports as an additive for professional training.

## Introduction

Analyzing and interpreting radiological examinations and documenting findings in written form is the key aspect of radiological work that has to be conveyed to younger colleagues during their residency^1–3^. Hence, continuous theoretical and practical training is paramount. However, the way residents access theoretical radiological knowledge has changed tremendously within the last decade. While radiological textbooks were considered a cornerstone of radiological expertise, this paradigm is challenged by the ongoing digitalization. While only 50% of the residents used a computer for offline database search in the 1990s, current surveys show that 99% of radiology residents primarily rely on online databases^4–6^.

Despite the advance of new media in radiological self-education, the impact of digitalization on practical training has been minimal until now. Here, primary image review and reporting are performed by a resident. Afterwards, a board-certified radiologist reviews preliminary reports to correct potential errors and provide continuous feedback to the reporting residents. Within this workflow, the so-called “radiology readout”, a mutual image reading session with experienced radiologists, is still a globally recognized tool to swiftly convey radiological knowledge to residents^7^. As the radiology report is a key clinical and legal﻿ component^8,9^ those read-out sessions are used to raise the quality of the residents' reports, too. Especially with regard to understandability, brevity, and overall impression of the reports, senior radiologists can share valuable insights with their younger colleagues^10^.

The acceptance of this teaching method, however, has continuously eroded due to an ever-increasing workload^11^. Additionally, the COVID-19 pandemic was an accelerator of this subtle process as it led to a gradual increase of radiologists working from home^12^. As this was considered very popular among radiologists it is unlikely that there will be a return to traditional work models even after the pandemic^13^. While greater availability of potential employees, improved work-life balance, and increased independence are advantages of remote working models, there are also significant drawbacks such as the challenges of integration into the clinical routine and communication with colleagues^14^. Especially, the loss of training opportunities is seen as a potential risk^14,15^. To counteract this, various concepts, such as the implementation of virtual read-out sessions for regular personal feedback or the provision of reporting curricula, have been introduced internationally and have been generally well-received^16–19^. Anyway, it is intriguing that young residents deliver a less positive of the virtual read-out compared to senior radiologists^20^. This data suggests that young residents' have an urgent need for very regular feedback. Furthermore, it is difficult for residents to perceive subtle changes in approved reports. Thus, the opportunity to obtain skills in conveying individual opinions in ambiguous findings, a key aspect of radiological reporting, is endangered. Here, the manual comparison of preliminary and approved reports is not only time-consuming but also prone to errors and therefore not feasible in everyday clinical practice. To address this problem, we developed an in-house software (DiffTool), to track changes made to preliminary reports by board-certified radiologists. The goal of the present study was to evaluate the acceptance and effectiveness of this software solution for the radiological training of residents.

## Methods

### Ethics statement

The local institutional review board (Ethics Commission of the University Duisburg-Essen, Germany) waived the ethics approval and informed consent for this pseudonymized employee attitude survey.

### Study design & questionnaire

The DiffTool software was developed to improve the education of residents. Prior to the software launch in 05/2022, information about the study habits and educational needs of residents at our department was collected anonymously via a structured online questionnaire (t_0_). The base questionnaire at t_0_ contained 17 questions. Primarily, the first eleven questions assessed the residents' sex, age, and radiological work experience in general and for each of the main three imaging modalities (radiography, CT, MRI) specifically. Questions 12–14 collected information on whether residents are tracking, understanding and learning (from) the changes made to their preliminary reports. With this data, we aimed to comprehensively evaluate the relevance of the report correction carried out by the board-certified radiologists. Questions 15 and 16 with a sub-set of five questions each analyzed the preferred modality to obtain radiological knowledge right now and their teaching needs regarding the remaining residency. Finally, the survey at t_0_ featured a statement aimed at evaluating the requirement for a new software solution for monitoring changes made to preliminary reports. After two to four months of use, data collection was repeated via the same questionnaire, along with additional questions regarding the DiffTool (t_2−4_) to ensure optimal comparability and assess any changes in user perspectives. In the questionnaire at t_2/4_, the user experience as well as the significance of the DiffTool for the daily routine of the residents was evaluated with three additional questions to understand the impact of the new software in clinical care. (See s Supplement 1 for the complete questionnaire).

Responses were recorded using a six-point Likert scale ranging from 1 (strongly agree) to 6 (strongly disagree, see supplementary material 1 for the complete questionnaire).

### Software

The DiffTool is a web-based application developed in-house without disrupting established workflows in radiological departments that allows users to compare and analyze differences between versions of diagnostic reports.

(Fig. 1).

**Figure 1:**
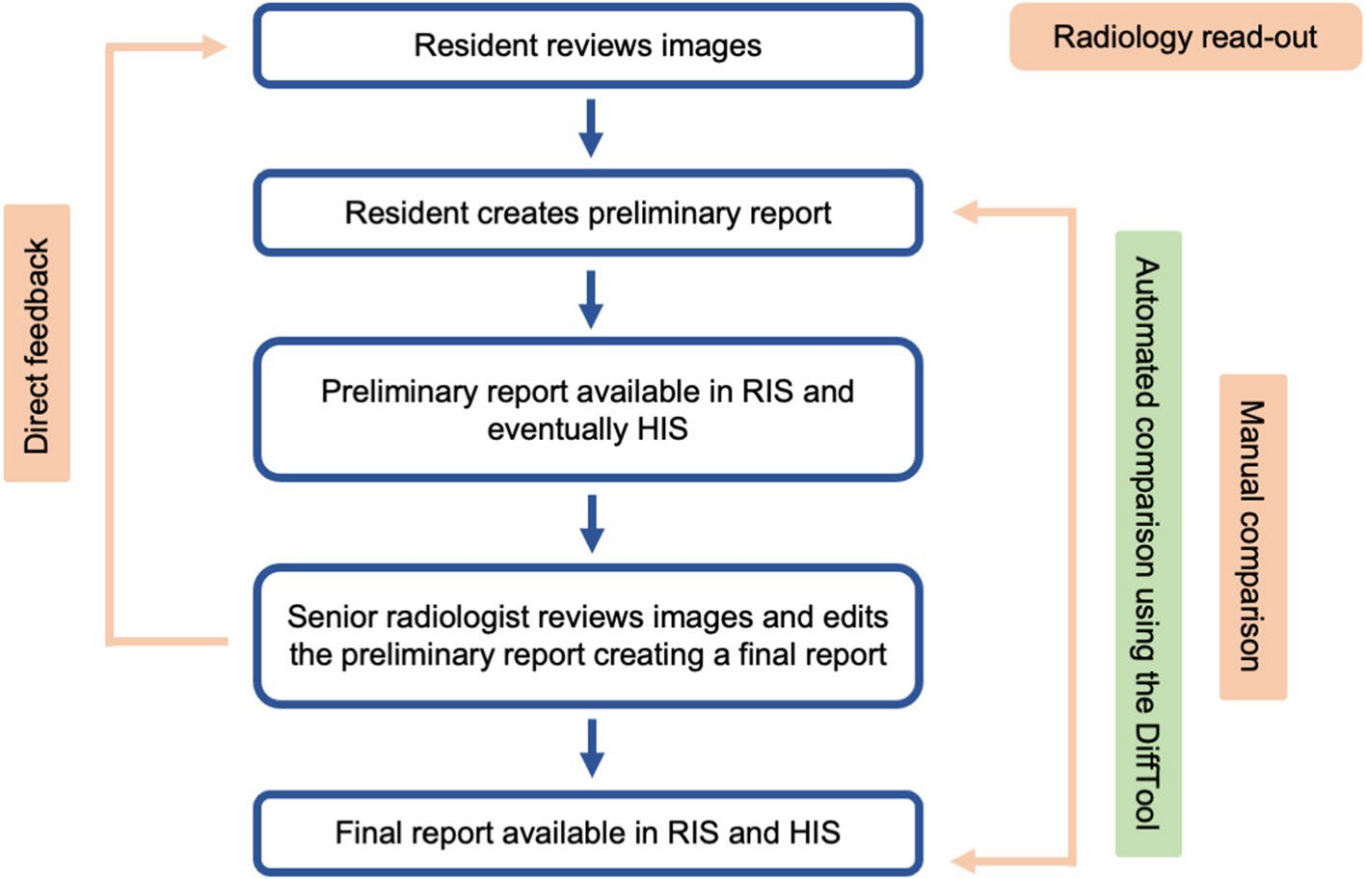
Workflow in a radiology department and possible teaching points. RIS = Radiology Information System, HIS = Hospital Information System.

It was built using TypeScript, React, and Scala and is based on the local Smart Hospital Information Platform (SHIP), which uses the Fast Healthcare Interoperability Resources (FHIR) standard to store and transfer medical data. SHIP offers a Representational State Transfer Application-Program-Interface-Type (REST API) that returns data as JavaScript Object Notation (JSON) objects. Various applications, including the DiffTool, have been developed to support patient care and research at the hospital. To access and manipulate data in SHIP, users must authenticate themselves using an existing SHIP app called Ship-auth, which creates a JSON web token (JWT). The DiffTool uses this SHIP token to validate user permissions and display a list of diagnostic reports for the current date or a specified date range. In this overview, details on the report status and the extent of changes in percent are visualized. By clicking on a report, it is possible to get a detailed comparison between different versions of the report. The software provides a multi-level comparison possibility in which different variants of the report can be compared (e.g. in hybrid imaging where different adjustments are made by a board-certified radiologist and a nuclear medicine physician). Users can then select two versions of a diagnostic report to compare, and the tool uses the react-diff-viewer library to highlight differences, including whitespaces, commas, deleted, changed, or added text. Differences between the selected versions are highlighted to give users a quick overview. Here light colors (either red or green) indicate additions while dark colors represent removed text segments.

**Figure 2:**
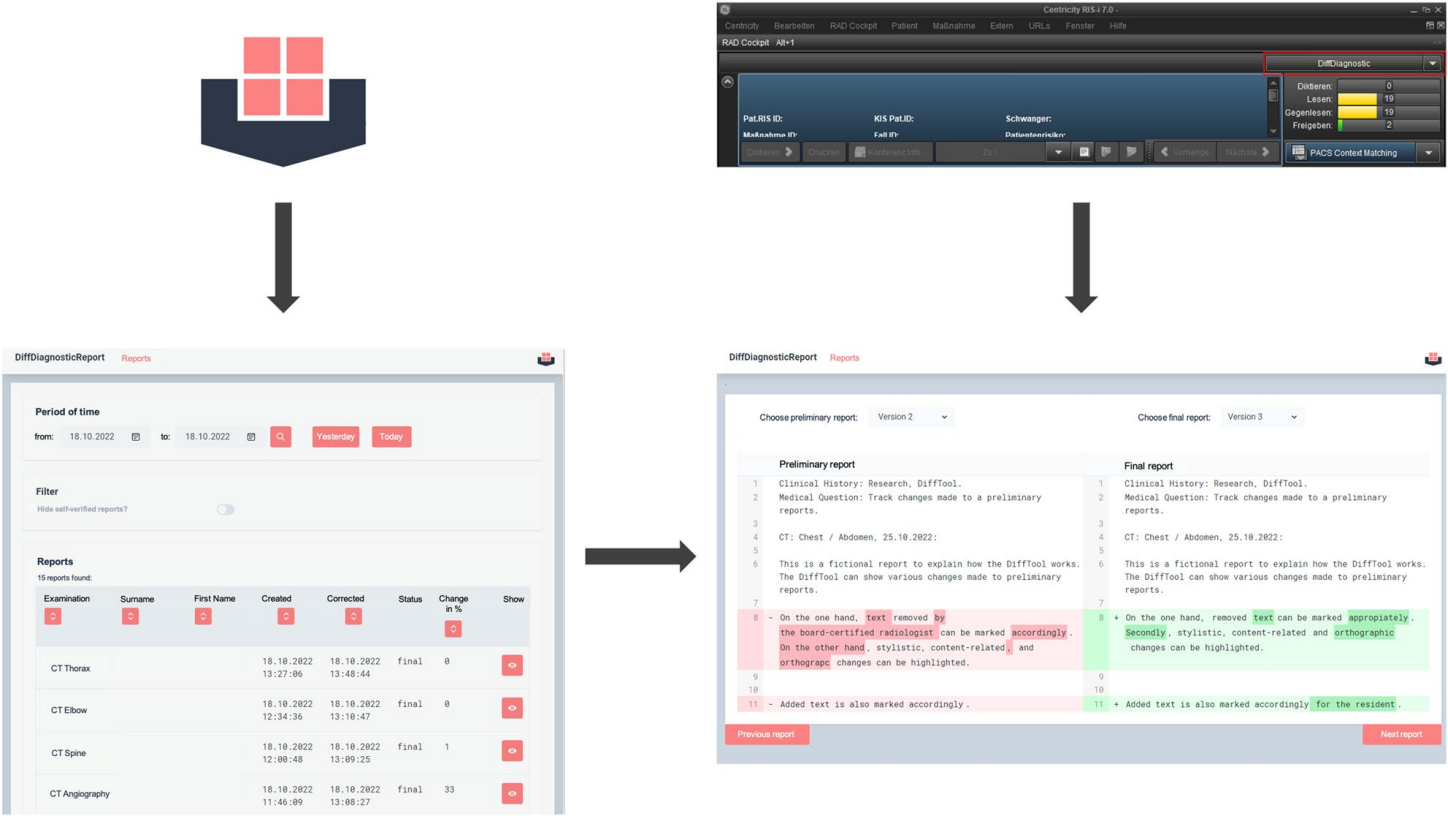
Overview of the user interface of the DiffTool.

The DiffTool retrieves the reports by sending GET requests to the SHIP-FHIR server with the report IDs and, in the case of the preliminary report, the keyword “history”. To retrieve the preliminary report, the last entry in the history should be selected. Users can also access the DiffTool by opening a case in the Radiology Information System (RIS) and clicking on a link to the DiffTool, which will then verify the user's credentials and search for the desired report. (Fig. 2).

### Statistics

Since only a limited number of residents participated in the study (n = 18) only explorative and observational data analysis was performed using GraphPad Prism version 9.4.1 for MacOS (Dotmatics, Boston, MA, USA).

### Definitions

To enhance reader comprehension, the number of specific responses was abbreviated according to the following scheme (R_x_ = number of responses), where x corresponds to the response on the Likert scale. The term “new media” can be confusing because it covers a broad spectrum of digital content, which may lead to different interpretations. When referring to "new media" in this article, we include a wide range of digital content, including websites, videos, learning platforms, and online databases. However, it's important to note that we exclude purely digitized texts like books and journals.

## Results

The study population was composed of 18 radiology residents of the radiological department at the investigating hospital. Their radiological experience ranged from one year to five years (median: 2 years; IQR: 1.25–3). Six residents were female and 12 residents were male, with a median age of 29 years (IQR: 28–29). All residents completed both questionnaires, and no dropouts were observed.

(Table 1).

**Table 1:** Demographics Table at t_0_.

Variable	Study population *(n* = *18)*
Age, years*	29 (28–29)
Sex, female	6 (33%)
Experience in radiology, years*	2 (1.25–3)
Experience in radiograph evaluation, month*	10.6 (6–23.25)
Experience in CT evaluation, month*	10.5 (1–18.5)
Experience in MRI evaluation, month*	1.5 (0–4.5)

* = Data are medians with interquartile range.

At t_0_, 22% (4/18) of the residents have primarily analyzed radiographs for the last four month while 72% (13/18) worked on CTs and 6% (1/18) on MRI. In line with this, we have also asked the residents about their confidence and level of knowledge with regard to the distinct imaging modalities. 78% (R_1_ + R_2_ = 14/18) agreed (strongly) on having acquired good knowledge with respect to the reporting of radiographs over the course of their residency, while 44% (R_1_ + R_2_ = 8/18) made the same statements about CT and 22% (R_1_ + R_2_ = 4/18) about MRI. Regarding the primary workplace of the last four month 72% (R_1_ + R_2_ = 13/18) (strongly) agreed on feeling confident in generating reports.

**Figure 3:**
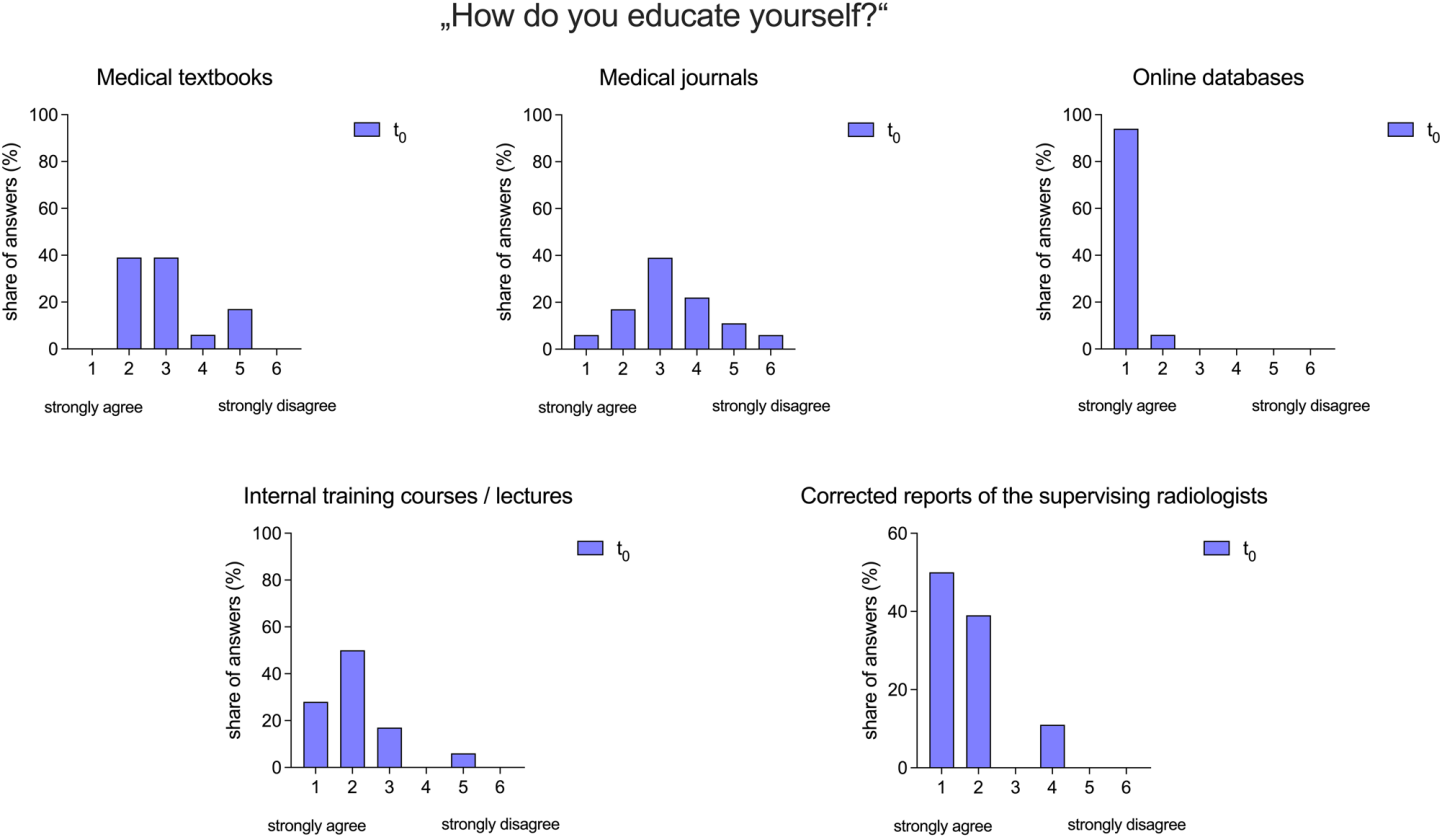
Opinion of radiology residents on the significance of various methods for radiological self-education. The importance of each method was assessed using a six-point Likert scale ranging from 1 (strongly agree) to 6 (strongly disagree).

Before the DiffTool was made available to the residents in 2022, we assessed the habits of the residents regarding their professional training. Residents unanimously (strongly) agreed on the significance of online databases on their radiological education (R_1_ + R_2_ = 18/18), while only 41% (R_1_ + R_2_ = 7/18) provided an identical response for medical textbooks and 22% (R_1_ + R_2_ = 4/18) echoed this for medical journals. However, internal training activities were still considered as important by 78% (R_1_ + R_2_ = 14/18) of residents. Additionally, 89% (R_1_ + R_2_ = 16/18) agreed on the importance of corrected radiology reports for their training. (Fig. 3).

In addition to their habits, we investigated the desire for other teaching methods of residents regarding their professional training. 78% (R_1_ = 14/18) strongly agreed with the statements that they would like to have access to online databases, internal training, and access to radiological conferences, respectively. 44% (R_1_ = 8/18) strongly agreed with the statement that they would like to have more access to medical textbooks and medical journals, respectively.

To evaluate the need for regular feedback in hands-on training, we evaluated the need and wish for a software tool to keep track of changes in finally approved reports. 61% (R_1_ = 11/18) of the residents strongly agreed with the statement: “I wish I had a software tool to keep track of changes made in my reports by my supervisors”.

(Fig. 4).

**Figure 4:**
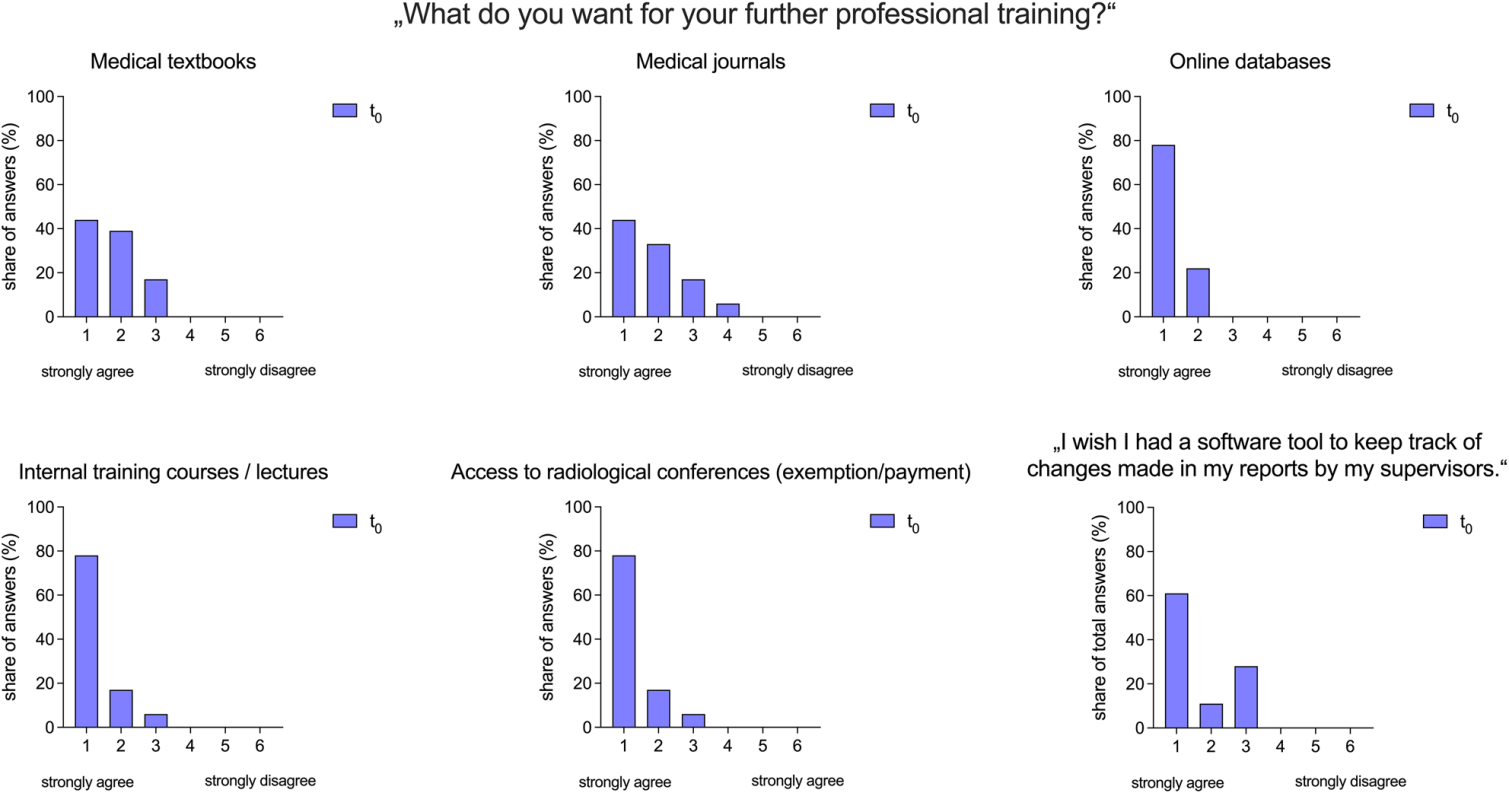
Desires of radiological residents for further teaching methods. The importance of each method was assessed using a six-point Likert scale ranging from 1 (strongly agree) to 6 (strongly disagree).

To circumvent a potential bias by the previous item, we also analyzed whether residents were manually tracking corrections made by board-certified radiologists in the preliminary reports of the residents. Prior to the deployment of the DiffTool 39% (R_1_ = 7/18) of residents reported to track corrections for every report. After the launch of the DiffTool, there was an increase from 39% (R_1_ = 7/18) to 61% (R_1_ = 11/18) of residents who strongly agreed on the statement that they tracked the changes made to every report. Although only a slight increase in residents strongly agreeing with the statement “I can understand the changes made by my supervisors in my reports” from 28% (R_1_ = 5/18) to 33% (R_1_ = 6/18) was observed, the software seems to facilitate the tracking of changes and therefore has a positive impact on residents. To evaluate whether the development of the DiffTool improved the radiology training we asked, whether residents used corrected reports for additional self-education. Two to four months after the deployment of the DiffTool 67% (R_1_ = 12/18) of the residents strongly agreed on that statement compared to 50% (R_1_ = 9/18) at t_0_. Additionally, we observed an increase from 33% (R_1_ = 6/18) to 44% (R_1_ = 8/18) of residents who strongly agreed with the statement “I profit from every corrected report”.

(Fig. 5).

**Figure 5:**
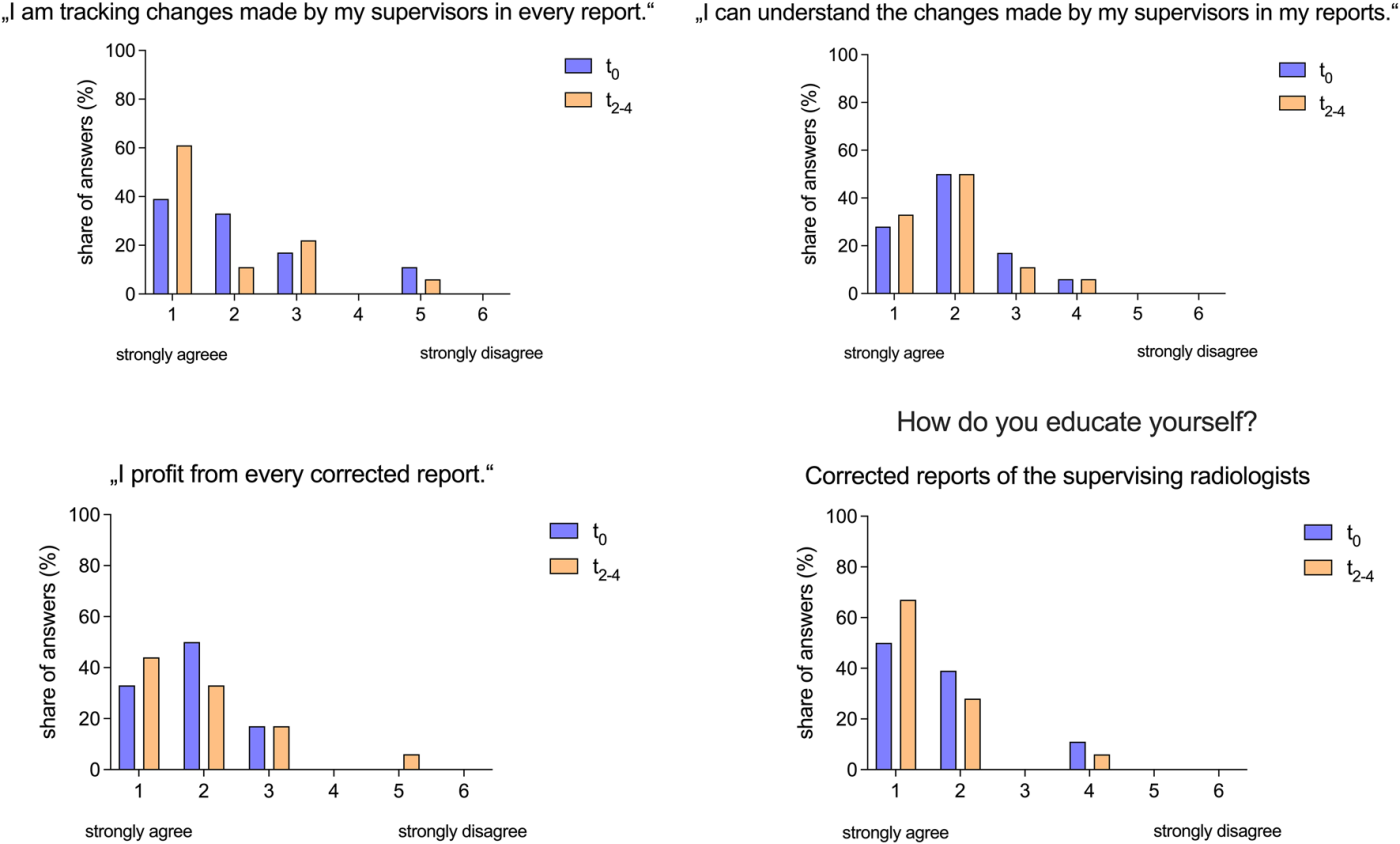
Impact of the DiffTool on tracking, understanding and learning from corrected reports. The importance of each statement was assessed using a six-point Likert scale ranging from 1 (strongly agree) to 6 (strongly disagree).

**Figure 6:**
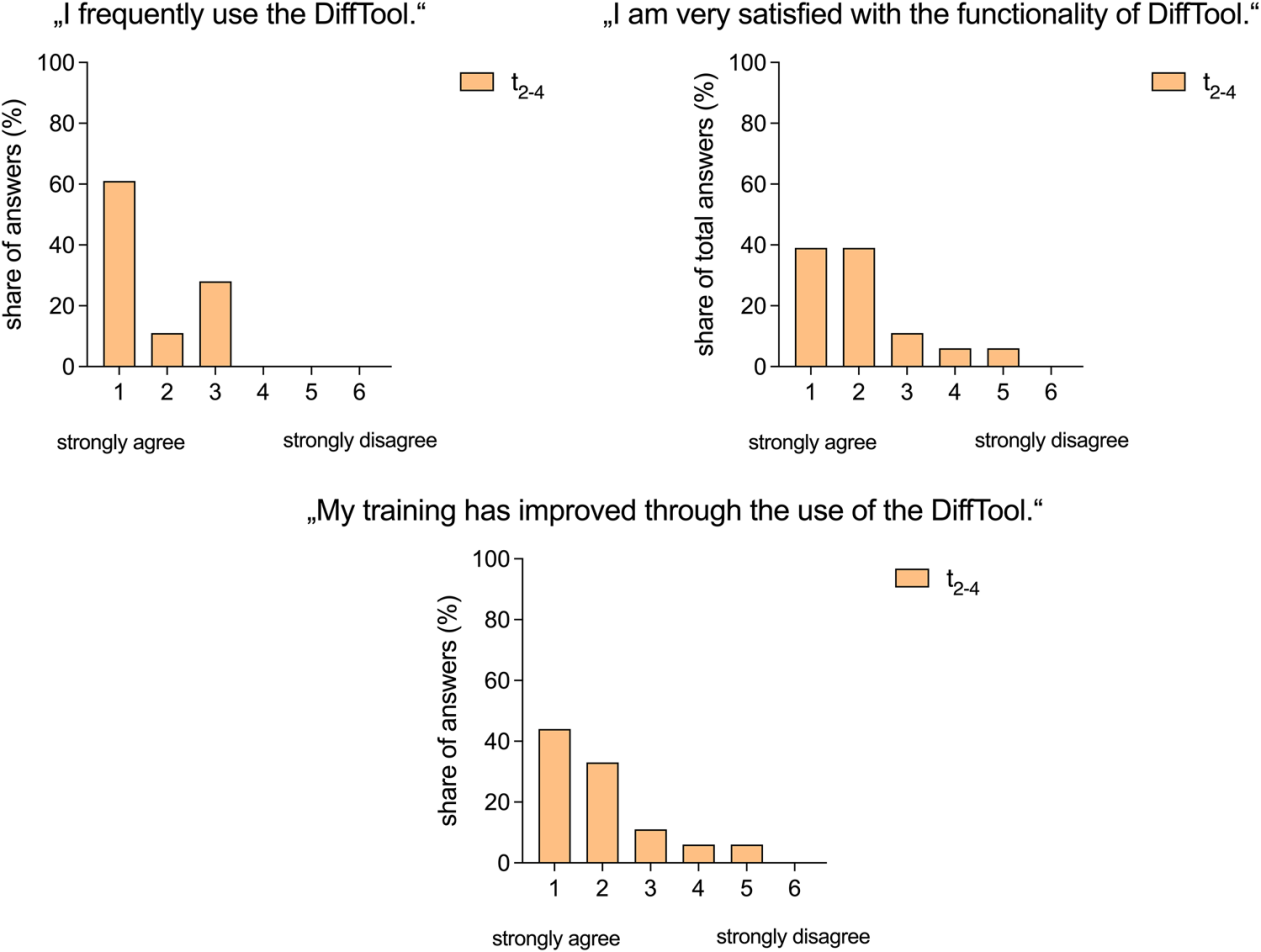
Evaluation of the overall user experience of the DiffTool. The importance of each statement was assessed using a six-point Likert scale ranging from 1 (strongly agree) to 6 (strongly disagree).

The software itself was well received by the residents. A majority of 72% (R_1_ + R_2_ = 13/18) agreed on using the DiffTool regularly. A majority of 78% (R_1_ + R_2_ = 14/18) agreed with the statement “I am very satisfied with the functionality of the DiffTool”. 78% of the residents (R_1_ + R_2_ = 14/18) agreed with the statement “My training improved through the use of the DiffTool. (Fig. 6).

## Discussion

Established tools in radiological hands-on training, such as radiology read-outs, are under continuous pressure due to the ever-increasing workload as well as ongoing challenges such as the COVID-19 pandemic or evolution to decentralized workspaces^12,13,21–24^. To address the residents´ needs, a thorough investigation of the current situation is necessary. Furthermore, we assessed an in-house developed software, DiffTool, to improve practical training by establishing a workflow for residents to track changes made to preliminary reports by senior radiologists. Our investigation revealed four major findings. First, new media, especially online databases, are central to professional education. Second, tracking reports of changes made by supervising radiologists is the second most important way for residents to improve their radiological education as 89% (strongly) agree (R_1_ + R_2_ = 16/18 at t0) on that statement (Fig. 3), which underscores the importance of report correction for training that has been shown before^25,26^. Third, the launch of DiffTool encouraged more residents to track their reports and raised awareness for this way of self-education. Fourth, we observed a broad adoption of this new teaching tool due to a satisfactory user experience leading to an improvement in the residents’ education.

On their way to the board examination, residents obtain radiological knowledge by self-study, lectures, or hands-on teaching. While most residents used medical textbooks for self-study in the past, we observed that in our cohort, online databases were considered the most important tool for residents to access radiological knowledge. These findings are in line with a recent survey by Derakhshani et al. revealing that new media, especially online databases, are the most important source of information for residents nowadays^5^. The outstanding role of online databases is matched by their recent user data. For instance, the open-access online library radiopaedia.org founded in 2005 noted a continuous incline to 20 million page-views per month in 2020^27^.

Although internal training courses or lectures were considered as less important than online databases, residents preferred these formats in direct comparison to textbooks or medical journals in our study. In accordance with these findings, the majority of residents expressed their wish to access online databases, congresses, and internal training to improve their radiological training. Here, the possibility to access online presentations at virtually any time from any place in the world might provide new teaching opportunities for residents, however, the benefit of these teaching concepts has to be elucidated in further studies. Despite the ever-growing importance of new media, access to medical books and journals is still considered relevant by the interviewed residents as 44% still strongly demand access to medical textbooks and journals, respectively. These findings are in line with the above-mentioned results from Derakhshani et al., who observed that medical journals were still considered an important secondary source in challenging cases^5^.

Although various options are available for radiological self-education thanks to the advancing digitalization in the early twenty-first century, direct hands-on teaching is fundamental and an internationally well-accepted method in radiological residency^7^. However, since the radiological report is a legally significant and clinically guiding document^8^, it is important to provide additional training in terms of structure, syntax, or brevity^10,28,29^.

As 89% of the residents (strongly) agreed with the statement “I profit from every corrected report”, regular, preferably daily feedback by an experienced radiologist is necessary. However, the possibility of providing such feedback in a common radiology readout session was minimized during the COVID-19 pandemic. Although these restrictions have been abolished, it is questionable whether the trend of remote reporting, which was kickstarted during the pandemic, will ever be fully reversed, given the positive feedback by radiologists^13,30,31^. Leaving the positive effects of remote working like better work-life balance apart, there is an undeniable risk of reduced face-to-face contact between employees^14^. Naturally, this also includes the interaction between board-certified and resident radiologists, which, in addition to the high workload, further diminishes one-on-one hands-on teaching opportunities^14,15^. Various studies have investigated the possibility of employing virtual radiology read-out sessions with positive feedback^17–19^. However, Matalon et al. have elucidated that remote feedback mechanisms can pose a particular threat to the education of junior residents and suggest that they require more feedback^20^. The results of our study indicate that such feedback does not exclusively have to be conveyed in a face-to-face conversation, as 61% of the residents demanded a software solution to track changes made by superiors as an addition to exsting feedback mechanisms. Indeed, the introduction of the software led to an increased awareness of corrections made in final radiology reports as a majority of 61% of the residents strongly agreed on tracking the corrections for every report, while only 39% did track those changes to that extent manually before. Additionally, a greater share of residents benefited from the implementation of the DiffTool as they improved their radiological knowledge with every corrected report (R_1_ after = 44%; before: 33%). However, the introduction of the DiffTool resulted only a modest increase in strong agreement regarding residents' understanding of changes in their reports, with only 33% at t_2/4_ (vs. t_0_ = 28%). This underscores the limitations of the DiffTool, as it cannot facilitate comprehension of alterations made. For the time being, in-person interaction remains of great significance. Additionally, an add-on to the DiffTool with annotation functionality for board-certified radiologists is worth considering in the future. Still, as the primary intention of the software was not comprehension improvement but raising the awareness of made alterations, it's worth noting that an encouraging majority of 83% at t_2/4_ (vs. t_0_ = 78%) either strongly agreed or agreed on understanding changes made to their preliminary reports. As multiple aspects within a radiological report can be subject to corrections, ranging from image interpretation to minor adjustments in syntax that impact the statement's accuracy, it becomes crucial to investigate the impact of software solutions like the DiffTool on specific error subgroups in the future.

These results support the findings of Sharpe and Kalaria et al. that indicated the potential of such software. At their department, residents checked for corrections made in their reports more frequently after the software launch and improved their radiological knowledge in that manner^25,26^. Unfortunately, their software solution could not be rolled out to other departments due to the lack of interoperability. Therefore, the DiffTool is based on international standards like FHIR to ensure an easy transfer to other institutions. Apart from the sole improvement to the training, the DiffTool software was well accepted among residents as 72% (strongly) agreed on using it regularly. Additionally, 78% were satisfied with the functionality of the DiffTool and stated that the software improved their radiological training significantly, a result echoing the positive evaluations reported by Kalaria et al^26^.

However, there are a few limitations to address in the present investigation. First, only a limited number of residents from one department participated in the study. Moreover, the software and its functionality were adjusted towards the workflow in the department of the investigating hospital. Because of the missing long-term follow-up and the difficulty in measuring the gain of radiological knowledge, there is a risk of a temporary novelty effect. However, we tackled the issue of generalizability by using standardized interfaces such as FHIR for easy translation to other systems.

In conclusion, the demand for direct feedback and hands-on teaching is still high despite new digital opportunities for radiological self-education. To satisfy this demand despite the ever-increasing number of home office workspaces kickstarted due to the COVID-19 pandemic, a novel software (DiffTool) was developed to track changes made to reports automatically. Thanks to the software, residents tracked reports more frequently and stated that the highly accepted, and considered this easy-to-use software a welcomed addition to their radiology training.

### Supplementary Information

Supplementary Information.

## Data Availability

Data, material and all necessary codes can be made available upon request via the corresponding author.
