# Supplementary material for: The importance of educational tools and a new software solution for visualizing and quantifying report correction in radiology training
Source: Sci Rep. 2024 Jan 12;14:1172. doi: 10.1038/s41598-024-51462-4 (PMC10786897; doi:10.1038/s41598-024-51462-4)
Supplement: Supplementary file 1 — Supplementary Information. [file 41598_2024_51462_MOESM1_ESM.docx]

The importance of educational tools and a new software solution for visualizing and quantifying report correction in radiology training

Luca Salhöfer^1,2*^, Johannes Haubold^1,2*^, Maurice Gutt^3^, René Hosch^2^, Lale Umutlu^1^, Mathias Meetschen^1,2^, Maximilian Schuessler^1,2^, Michael Forsting^1^, Felix Nensa^☨1,2^, Benedikt Michael Schaarschmidt^☨1^

^1^ Institute of Diagnostic and Interventional Radiology and Neuroradiology, University Hospital Essen, Essen, Germany

^2^ Institute for Artificial Intelligence in Medicine, University Hospital Essen, Essen, Germany

^3^ Central IT services, University Hospital Essen, Essen, Germany

^*,☨^ The authors/supervisors contributed equally to this work.

## Supplementary Material

### Supplementary Material 1: Online Questionnaire

Answers for the questions 1 - 6 were collected as free text. For the remaining questions, answers were obtained using a six-point Likert scale ranging from 1 (strongly agree) to 6 (strongly disagree).

1. Sex
2. Age
3. What was your main workplace in the last 4 months?
4. Year of residency?
5. What is your experience in months regarding the evaluation of radiographs?
6. Experience in month regarding CT evaluation?
7. Experience in month regarding MRI evaluation?
8. I have acquired good knowledge in the reporting of radiographic examinations during my residency.
9. I have acquired good knowledge in the reporting of CT examinations during my residency.
10. I have acquired good knowledge in the reporting of MRI examinations during my residency.
11. I feel confident in writing radiological reports during the last four months.
12. I am tracking changes made by my supervisors in every report.
13. I can understand the changes made by my supervisors in my reports.
14. I profit from every corrected report.
15. How do you educate yourself?
    1. Medical textbooks
    2. Medical journals
    3. Online databases
    4. Internal training courses / lectures
    5. Corrected reports of the supervising radiologists
16. What training methods would you like to use in the future to improve your radiological training?
    1. Access to online databases
    2. Access to radiological conferences (exemption/payment)
    3. Internal training courses / lectures
    4. Access to medical textbooks
    5. Access to medical journals
17. I wish I had a software tool to keep track of changes made in my reports by my supervisors.

The following questions were only part of the second survey at t_2-4_:

1. I frequently use the DiffTool.
2. I am very satisfied with the functionality of DiffTool.
3. My training has improved through the use of the DiffTool.
